# Improving Prosthetic Selection and Predicting BMD from Biometric Measurements in Patients Receiving Total Hip Arthroplasty

**DOI:** 10.3390/diagnostics10100815

**Published:** 2020-10-14

**Authors:** Carlo Ricciardi, Halldór Jónsson, Deborah Jacob, Giovanni Improta, Marco Recenti, Magnús Kjartan Gíslason, Giuseppe Cesarelli, Luca Esposito, Vincenzo Minutolo, Paolo Bifulco, Paolo Gargiulo

**Affiliations:** 1 Department of Advanced Biomedical Sciences, University Hospital of Naples ‘Federico II’, 80131 Naples, Italy; 2 Institute for Biomedical and Neural Engineering, Reykjavík University, 102 Reykjavík, Iceland; dcrjacob@gmail.com (D.J.); marco18@ru.is (M.R.); magnuskg@ru.is (M.K.G.); paologar@landspitali.is (P.G.); 3 Faculty of Medicine, University of Iceland, 102 Reykjavík, Iceland; halldor@landspitali.is; 4 Landspítali Hospital, Orthopaedic Clinic, 102 Reykjavík, Iceland; 5 Department of Public Health, University Hospital of Naples ‘Federico II’, 80125 Naples, Italy; ing.improta@gmail.com; 6 Department of Chemical, Materials and Production Engineering, University of Naples “Federico II”, 80125 Naples, Italy; giuseppe.cesarelli@unina.it; 7 Istituto Italiano di Tecnologia, 80125 Naples, Italy; 8 Department Engineering, University of Campania Luigi Vanvitelli, 81100 Aversa (CE), Italy; luca.esposito@unicampania.it (L.E.); vincenzo.minutolo@unicampania.it (V.M.); 9 Department of Electrical Engineering and Information Technologies, University Hospital of Naples ‘Federico II’, 80125 Naples, Italy; pabifulc@unina.it; 10 Department of Science, Landspítali Hospital, 102 Reykjavík, Iceland

**Keywords:** database analyses, electromyography, machine learning, clinical decision making, total hip arthroplasty

## Abstract

There are two surgical approaches to performing total hip arthroplasty (THA): a cemented or uncemented type of prosthesis. The choice is usually based on the experience of the orthopaedic surgeon and on parameters such as the age and gender of the patient. Using machine learning (ML) techniques on quantitative biomechanical and bone quality data extracted from computed tomography, electromyography and gait analysis, the aim of this paper was, firstly, to help clinicians use patient-specific biomarkers from diagnostic exams in the prosthetic decision-making process. The second aim was to evaluate patient long-term outcomes by predicting the bone mineral density (BMD) of the proximal and distal parts of the femur using advanced image processing analysis techniques and ML. The ML analyses were performed on diagnostic patient data extracted from a national database of 51 THA patients using the Knime analytics platform. The classification analysis achieved 93% accuracy in choosing the type of prosthesis; the regression analysis on the BMD data showed a coefficient of determination of about 0.6. The start and stop of the electromyographic signals were identified as the best predictors. This study shows a patient-specific approach could be helpful in the decision-making process and provide clinicians with information regarding the follow up of patients.

## 1. Introduction

Advanced arthroplasty and pain in hip joints have been extensively treated with total hip arthroplasty (THA). For several years, hip replacement with a prosthesis has been among the most effective orthopedic surgeries for re-establishing function and lowering the pain of patients affected by various pathologies, particularly osteoarthritis and osteonecrosis of the femoral head and neck fractures. Patients generally experience less pain in the months and years following a THA or hip resurfacing procedure; nevertheless, many of them express disappointment with their capacity to perform daily tasks, a reduced ability to walk and long-term muscle weakness in the lower extremities.

THA can be performed with or without the use of bone cement. The advantage of a cemented procedure is that stability can be achieved earlier in the recovery process than with an uncemented procedure, in which the primary implant stability is obtained through a geometrical fitting, press-fit forces and friction between the bone and implant, while the secondary stability is achieved by bone ingrowth into the surface texture of the femoral component. However, use of the cement technique can make the local bone weaken as a result of decreased mechanical stresses; that is, the density of the bone progressively declines around the prosthesis, resulting in an increased fracture risk. Moreover, bone resorption is recognized as one of the most common causes for the loosening of the stem [1]. In the case of an uncemented prosthesis, surgery is performed using a press fit, and the stratum of the bone directly neighboring the implant stem is preloaded and stimulated to grow [2]. In the first few years following surgery, uncemented stems receive more revisions than cemented ones because of periprosthetic fractures, but the risk of infection is similar between the two options [3]. On the one hand, the management of these fractures is a difficult task for surgeons because of the low quality of the adjacent bone [4]. On the other hand, revision operations for uncemented implants achieve a higher success rate and generally lead to fewer complications than revision surgeries for cemented implants [5,6,7], in which issues are also linked to the risk of cement cracking because of fatigue. Nevertheless, the overall survival rate of uncemented THA is still marginally inferior to that of cemented THA according to the Swedish Hip Registry [8]. In order to recommend an uncemented prosthesis, the patient’s bone quality must be assessed: the femur must resist the press fitting and compressive stress during surgery and during consequent functional loading. The bone ingrowth should also be predictable. Currently, a common decision criterion for indicating whether to employ a cemented or an uncemented prosthesis does not exist. Different results have been found by important studies, but a consensus has not yet been reached [7]. Clinicians have to choose between the two types of prosthesis according to the personal situation of each patient. Measurable preoperative evaluations of bone quality are not taken into account with the existing guidelines, even though it is well known that they would have a positive effect on the success of the surgeries [8]. Because the mineralization of bone diminishes with aging, cemented arthroplasty is, in general, more commonly chosen for older, inactive people and those with weak bones; uncemented replacements are more commonly chosen for younger and more physically active people [9]. Because the probability of the need for revision surgery increases with age, patients receiving an implant early in life have a higher probability of needing revision surgery, and the risk of intra-operative fracture under press fitting due to poor bone quality is lower in the population [10,11].

In the decision-making process regarding uncemented or cemented THA for the individual patient, bone and muscle quality are regularly included, when, for example, the biological age of the patient is estimated. Preoperative measurements of bone and muscle quality are not performed routinely in the clinic today, despite it being commonly accepted that they can have decisive influence on the outcome. Although age is one indicator for bone and muscle quality, individual differences due to lifestyle and genetics are wide, providing a broad range of bone density and muscle quality [12], which should be taken into consideration. Gargiulo et al. proved statistically that the quantitative measurements of both bone and muscle quality would certainly provide valid support to clinicians in their decision making [13].

### 1.1. An Introduction to the Methodology

THA has been investigated with different methodologies and from various points of view [14,15]: Lean Six Sigma was used to improve the process of THA and total knee arthroplasty in a university hospital [16,17]; artificial intelligence perfectly fits this context through machine learning (ML) techniques. ML is a subset of artificial intelligence and consists of the capacity of a computer to learn from training/past data in order to make predictions, and classification or regression analyses on new/test data. Many applications are present in the literature representing the implementation of ML algorithms with healthcare data: Ricciardi et al. applied it to study fetal well-being [18], Stanzione et al. and Romeo et al. applied ML in radiomics processes [19,20], and Ricciardi et al. performed ML studies for the diagnosis and prognosis of patients affected by coronary artery disease [21,22]. Haeberle et al., recently, conducted a review of ML in the orthopedic field, as well as Cabitza et al., who stated that there has been a 10-fold increase in reports mentioning ML in the last 20 years [23,24]. Different applications have been found in the orthopedic field and, especially, for the lower extremities: image-based analysis, mobile health technologies and value-based patients’ models. The aim of image-based analysis is to analyze, directly, images to detect and confirm, for example, the presence of osteoarthritis. Importantly, the number of mobiles has been increasing as well as the possibility to monitor remotely patients with their own mobile phones [25]; thus, ad hoc software could be developed for this purpose. Value-based patients’ models are an option that is considered in this paper: not only can ML detect pathologies or be used for classification, but it can also be employed to introduce a data-driven approach that helps the clinician implement personalized medicine, as modeled by a recent study from Navarro et al. [26].

In the literature, there are some reports of applications of machine learning to THA patients. Ramkumar et al. developed and validated a predictive machine learning model using preoperative patient demographics for estimating the length of stay after primary THA as the first step in identifying a patient-specific payment model [27,28], while Harris et al. developed predictive models for mortality and major complications after elective total joint arthroplasty that can be used to inform preoperative discussions and decisions in diverse healthcare settings [29]. However, there is also evidence from literature reviews that the advancement and employment of ML provides the opportunity to provide data-driven, high-performance medicine that can rapidly improve the knowledge, economics and delivery of lower extremity arthroplasty [23].

### 1.2. Aim of the Work

Considering the general lack of knowledge concerning the joint motion of the lower extremities in the THA population, there is a need to characterize the joint mechanics of people that have undergone THA so that interventions and rehabilitation strategies can be designed to reduce postoperative complications and improve mobility. The final goal could be the creation of a tool so that the management of these patients could be improved by providing them with the best option based on quantitative patient-specific data.

The aim of this paper was to employ ML techniques on data such as measurements of bone and limb function as evaluated by computed tomography (CT) scans, gait analysis, electromyography (EMG) evaluations and 3D geometric modeling to help clinicians in the decision-making process of choosing whether a cemented or an uncemented prosthesis is the best for a patient, thus creating a patient-specific evaluation. The acquisition and the elaboration of the data strictly follows the research of Gargiulo et al., meaning that all the procedures for acquiring data and obtaining parameters (such as those from gait analysis of EMG) are the same of those in previous research [15]. Recently, Recenti et al. performed a first implementation of logistic regression and k-nearest neighbor analysis in order to predict the bone mineral density (BMD) of patients undergoing THA using only gait analysis features [30].

In this paper, the first analysis is described to provide clinicians with suggestions regarding the best choice of prosthesis according to patient-specific data, while the second part presents a regression analysis of the follow up, similarly to Recenti et al. [30], namely, trying to predict the adaptation of the prosthesis to the bone of the patient according to BMD measures through tree-based algorithms that have not been used previously.

## 2. Materials and Methods

### 2.1. The Dataset

The cohort for this study includes 51 primary THA volunteer patients as part of a national database at the Icelandic National Hospital (LSH, Landspítali, Reykjavík, Iceland). All patients underwent primary and unilateral THA (the surgeon decided the kind of prosthesis) that allowed us to have a healthy leg for comparison. Both types of hip prosthesis were Zimmer (Hip Replacement products, Warsaw, IN, USA) (http://www.zimmer.com/medical-professionals/products/hip.html). All patients underwent a biomechanical assessment at the health facility 1–2 days before and 1 year after surgery. The mean age of the analyzed patients was 60.1 ± 10.6 years; males represented the 42.3% of the total sample. The Icelandic Bioethics committee approved the patient pre- and postoperative assessments. Application number: 13-127-S1, approved on 12 July 2013; Study type and level of evidence: ‘‘Level 2 prospective cohort study’’.

### 2.2. Gait Analysis and EMG Assessment

The patients underwent gait analysis assessment at the Grensás rehabilitation clinic, 6 weeks before and 52 weeks after surgery. In this study, KINE view, GAITRite and KINE EMG technologies, which acquired the EMG at 1600 Hz, were used.

**Figure 1:**
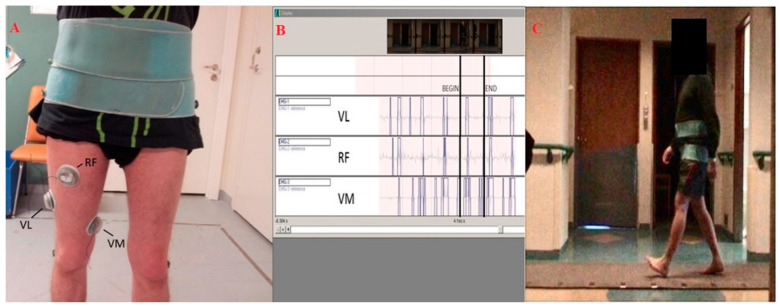
Acquisition of electromyography (EMG) parameters. Starting from the left, the panels show (**A**) the position of the electrodes for the EMG, (**B**) the EMG signals, and (**C**) a patient walking off the platform for the gait analysis. RF = Rectus Femoris, VL = Vastus Lateralis, VM = Vastus Medialis.

KineView employs advanced video capturing and data processing for patient gait using markers placed on the ankles, knees and hips. Wireless EMG probes are positioned on the vastus medialis (VM), vastus lateralis (VL) and rectus femoris (RFe). Muscle activation (start and stop, measured in time, in order to identify the timing of each muscle activation during the gait cycle) is visualized on different channels and automatically synchronized with video. The gait of the patients was recorded on an electronic walkway system, called GAITRite. This system is composed of more than fifteen thousand pressure switches set between two sheets of vinyl, which allowed us to measure many spatial and temporal parameters. These parameters and the on and the off of the muscles’ activity are used as input for the ML algorithms. Figure 1 represents the gait analysis and EMG assessment.

### 2.3. Imaging Acquisition

The procedure of the analyses was as that for those performed by Gargiulo et al. [13]. All patients were scanned with the same 64-slice spiral CT Philips Brilliance three times in one year: before surgery, immediately after surgery and, finally, at 52 weeks post-surgery. The CT scanned a region starting from the iliac crest and ending at the middle of the femur; the thickness of the slices was 1 mm, with a slice increment of 0.5 mm, and the voltage of the tube was fixed to 120KVp. These CT scans allow an accurate 3D reconstruction of the regions of interest.

### 2.4. Muscle and Bone Mineral Density Calculation

The segmentation of the images was performed with the MIMICS software (Materialise 20, Leuven, Belgium) (http://www.materialize.com/en/medical/software/mimics) to accomplish a separate analysis of the quadriceps muscles (RFe, VM and VL). Immediately and one year after the operation, the mean values of the Hounsfield Units (HU) for each muscle were computed.

**Figure 2:**
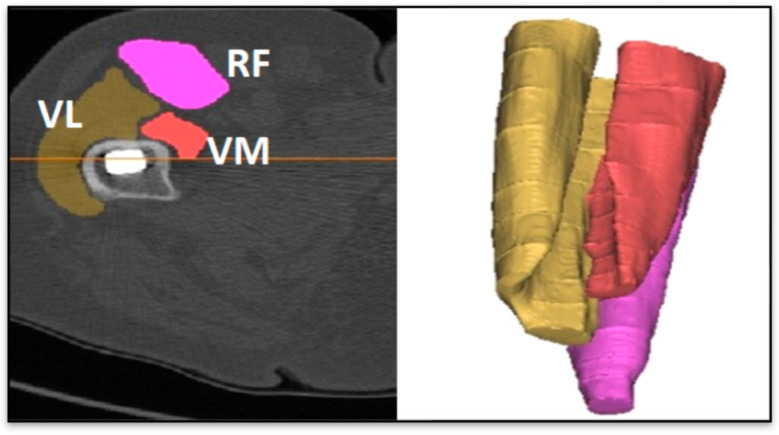
Segmentation of muscles in MIMICS: vastus medialis (VM) in red, vastus lateralis (VL) in gold and rectus femoris (RF only in the figure) in purple.

Variations in load response can cause bone remodeling according to Wolff’s law [31,32], where the BMD rises or declines, depending on the load magnitude and implant fixation method. Then, because one of the characteristics of THA patients is a decreased periprosthetic BMD due to the transmission of the stresses through the prosthesis and not through the bone [33], the process of bone remodeling in a time range of 52 weeks was mapped: the mean values of the BMD were computed from the CT scan in the proximal and distal femoral regions as shown in Figure 2, as mean values from segmented regions of interest. We used the same CT scanner for all the acquisitions, the scanner was pre-calibrated with the Quasar phantom from Modus Medical Devices Inc. (1570 North Routledge Park London, N6H 5L6 Canada) (www.modusmed.com) before each acquisition period. The Quasar phantom contains five different elements with known physical density. The measured HU values of the phantom’s five elements are plotted versus their physical density. We estimated a maximum difference, between the years, of 4.2% in the HU values within the muscle regions (from 20 to 90 HU); this deviation was considered in the calculation of the muscle mean densities. To accurately establish the relationship between the BMD and HU of the images from the CT scan, the elements in the phantom have a linear relationship above 0 HU and below 0 HU. When those linear relationships are united, the equation of the linear regression for the BMD–HU transformation is available. The Equation (1) we used to convert HU into BMD is the following:

BMD [g/cm^3^] = −8 × 10^−8^ × HU^2^ + 0.0006 × HU + 0.9456. (1)

The correlation coefficient for this calibration was R^2^ = 0.99.

**Figure 3:**
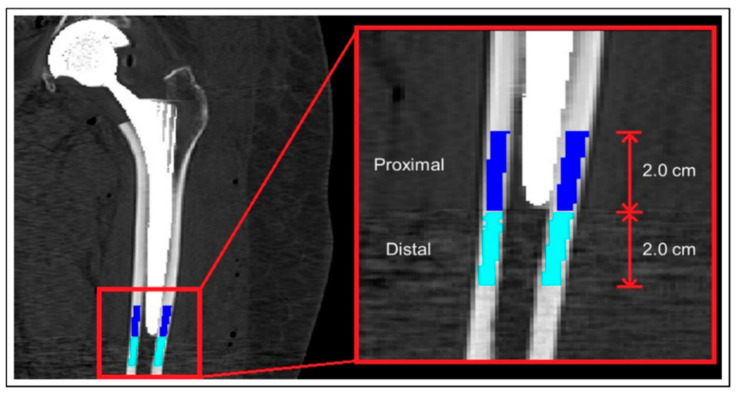
The distal and the proximal regions of the femur bone: the dark blue indicates 2.0 cm of the proximal part of the femur, while the light blue indicates the distal part.

The BMD of the bones (operated and healthy) and the HU of the muscles are used as input in the ML analysis. A representation of the analysis described in this paragraph is provided in Figure 2. Another model, ranging from the periprosthetic femur, without the femoral head (Figure 3), to the greater trochanter, with a distal axial cut through the lesser trochanter, was used to compute the BMD.

### 2.5. Tools and Algorithms

Several tools are available to help different users in decision making; however, in this study, the Knime analytics platform was chosen because it is a well-known platform, designed for advanced users [34] and employed in many biomedical studies [35,36,37]. This platform allows the creation of workflows by combining nodes that handle the different steps of an ML analysis. The algorithms implemented in this study were Random Forests (RF) and Gradient boosted tree (GB) to perform the classification for cemented or uncemented prosthesis. The same algorithms were also implemented to perform the regression analysis to evaluate the adaptation of the prosthesis on the bone (particularly, the BMD of the proximal and the distal region of the femur). Both analyses were supervised and needed a training and a testing phase: the classification consists of using some features to distinguish two or more different classes (in this paper, cemented and uncemented prostheses); the regression analysis is similar to the previous one as regards the training and testing phases, but it consists of predicting a continuous number (in this paper, the BMD of the distal and the proximal parts of the femur). GB and RF are both based on decision tree, a famous state-of-the-art structure made up of leaves and nodes, and exploit ensemble learning techniques: randomization, bagging and boosting. RF uses randomization and bagging, while the GB adds a form of boosting. RF operates by building a group of decision trees during the training time and outputting the class that is the mode of the classes (classification) or mean prediction (regression) of the individual trees, while GB is a generalization of boosting the arbitrary differentiable loss function; it gives greater weight to the records that are misclassified.

The Synthetic Minority Oversampling Technique (SMOTE) was slightly applied in order to balance the dataset [38]. It produces artificial data by extrapolating between a real record of an assumed class and one of the nearest neighbors of the same class.

### 2.6. Metrics and Workflow of the Machine Learning Analysis

The hold out was used to divide the dataset into training and test set (75% and 25%), the Wrapper method was employed to find the best subset of features maximizing the accuracy in the training set, and a leave-one-out cross validation was implemented to compute more-honest evaluation metrics [39]. The following were used for the classification analysis:

- Accuracy: the number of correct predictions over the total.
- Precision: a measure of the positive patterns correctly predicted from the total predicted patterns in a positive class.
- Recall: a measure of the fraction of positive patterns that are correctly classified.
- Specificity: better known as the “true negative rate”.
- Sensitivity: better known as the “true positive rate”.

The Area Under the Curve Receiver Operating Characteristics (AUCROC) were employed since they are a quantitative evaluator of binary classification.

The coefficient of determination (R^2^), mean absolute error, mean squared error, root mean squared deviation and mean signed difference were used for evaluating the regression analysis.

It is important to note that the algorithms were trained and tested on past clinicians’ decisions.

As regards the ML analysis, firstly, a procedure to handle missing data was implemented (some features used as input to the algorithms had a missing value, and we proceeded with missing data management to avoid losing an entire patient); all the missing data were replaced with the mean value for the considered feature. Then, SMOTE was applied, making the number of records increase to 54 (only 3 were added) to allow the exploitation of the whole power of tree-based algorithms, which need two balanced classes. After these pre-elaborations, the hold out split the dataset into training and test sets (respectively, 75% and 25% of the dataset); the wrapper was applied on the training set in order to find the best features to maximize the accuracy. The features identified by the wrapper method were used on the test set together with a leave-one-out cross validation. Finally, the evaluation metrics were computed.

## 3. Results

### 3.1. Classification Task

The assessment of the classification performance for both the training and test sets is shown in Table 1, while the selected features are shown in Table 2.

While the RF method obtained the highest results with the training set, the best algorithm was GB considering the test set, with an accuracy of 92.9%, a specificity of 100% and a good AUCROC (0.857). It was surprising that RF obtained such low results compared with the GB.

From Table 2, it is clear that the majority of the features helping in classifying patients into cemented or uncemented groups were related to the start and the stop of the EMG signal.

### 3.2. Regression Task

Firstly, the missing data were managed as they were in the previous task. In the regression analysis, the aim was to collect information on the follow up of the patients through a prediction: since the stresses on the proximal and distal parts of the femur are a good way to understand the reactions of the bone to the prosthesis, the BMDs of “proximal” and “distal” were the targets of the regression analysis. A matrix of correlation was computed, and a threshold of 0.6 was chosen: all the features overcoming this threshold were excluded since they did not add information to the classifiers. This threshold indicated a value beyond which the correlation can be considered strong and influential. A hold out method was employed, also, in this case to divide the dataset into a training and a test set. Finally, the evaluation metrics were computed. Table 3 and Table 4 represent the evaluation metrics and the features included in the algorithms.

All types of error were lower than 3%, and the R2 was mostly about 0.60. The majority of the selected features were related to the start and stop of the EMG. Moreover, it is interesting that the type of prosthesis, which was the target of the first analysis, was included in the feature selection of the regression analysis for the follow up.

## 4. Discussion

In recent years, the decision to employ a cemented or an uncemented prosthesis has been based only on the experience of the orthopedic surgeon and on the age and gender of the patients. In this study, a patient-specific approach using ML techniques and quantitative features was performed. First, after identifying the best subset of features through the wrapper method, a classification was performed for cemented or uncemented prosthesis. Second, the adaptation of the prosthesis to the femur after a year from the surgery was predicted through a regression analysis considering the BMD of the proximal and distal parts of the bone.

The results obtained in the classification task are important because they allowed the researchers to obtain two fundamental pieces of information. The first is well described by the evaluation metrics in Table 1; the accuracy of 93% is a valuable score, as well as the AUCROC of 0.857, and represents the validity of the approach. The second is shown by Table 2, as it represents the quantitative features that were identified as the most relevant in the study.

The results of the study are in agreement with clinical findings stating that the muscle mechanics play a key role in the success of the THA operation in terms of both strength [40] and temporal parameters during gait [41]. From Table 4, it can be seen how the algorithm identified the muscle parameters as among the most important, both in terms of temporal behavior during gait but also with regards to the HU values that can be correlated to the muscle quality and strength.

The results regarding the regression task had lower scores but are as relevant as the previous classification analysis. Not only would this approach allow the clinicians to receive a suggestion based on quantitative features regarding the type of prosthesis, but it would also provide them with a quantitative insight on the follow up of patients.

Before this study had been performed, only Gargiulo et al. had conducted some quantitative measurements of both bone and muscle to investigate in the same directions of this research [13,42,43]. The novelty of this study lies in two things: it is the first study aiming to help orthopedics in the decision-making process regarding the kind of prosthesis. Moreover, it is the first time that the decision regarding the kind of prosthesis has been based on a patient-specific approach, namely, by using measurements of bone and muscle density and function obtained by CT scans, gait analysis, the start and stop of EMG signals, and 3D geometric modeling. The addition of Finite Element Analysis (FEA)-based data, obtained by means of the automated construction of a numerical model from the cohort of patients’ CT data [44,45,46], could improve the proposed methodology by taking into account measures of punctual stress before and after the implant [47,48,49,50].

Compared with the previous pilot study [30], a new concept was introduced (namely, the classification for the type of prosthesis) and an extension of the algorithms and the features to predict the BMD was performed (namely, the features regarding the measurements of bone and function by CT scans and the EMG evaluations). These analyses fit with the idea of Haeberle et al., who considered ML not only for the classification of images but also as a valuable tool for moving in the direction of personalized care [23].

It is certain that increased mobility following THA is highly associated with better health status, physical activity levels [51], healthy body weights and improved quality of life [52]. However, the existing clinical preoperative assessment for THA patients does not employ the use of patient-specific gait profiles, which quantitatively characterize the quality and function of lower extremity muscles in recovering patients.

The purpose of this study was to compute data and information regarding the biomechanical and clinical aspects of THA and demonstrate the utility of ML technologies for supporting clinical decision making and post-operational assessment. Currently, THA surgical revision rates are increasing [53] and the effects of non-optimal prosthesis selections and fixation methods may dramatically affect patient mobility. These results demonstrate that patient-specific information such as gait parameters, BMD, and fracture risk calculation during press fitting are important in assessing patient conditions pre-THA and determining an optimal surgical strategy.

### Limitations and Future Development

There are some limitations to this study: the algorithms were trained on the past decisions made by clinicians regarding the type of prosthesis. Of course, the reader could wonder if this computation effort would be valid with a different decision of the clinicians. The question would be understandable, but this study aimed to investigate the feasibility of creating a new approach in this context: deciding the prosthesis of patients undergoing THA based on their quantitative data, thus following a patient-specific approach. Indeed, the algorithms could have also been trained on decisions made after an FEA analysis regarding the risk fracture for the bone, which might have contradicted the decisions of the clinicians. Therefore, a new future analysis could be based on the decision regarding the type of prosthesis based on the risk of fracture computed through FEA analysis, which is the direction recently embraced by some researchers [54].

## 5. Conclusions

This study proved that a patient-specific approach based on biometrics and ML technology can effectively support implant strategy decision making in THA surgery. Particularly important for indicating cemented or uncemented prosthesis are the skeletal muscle parameters such as the start and stop of muscle contraction from EMG signals and temporal and spatial gait parameters. Moreover, the regression analysis showed that employing machine learning could also provide clinicians with information regarding the follow up of patients, namely, predicting the BMD of the distal and proximal parts of the operated femur after one year from the surgery. Future studies should focus on implementing tools with these features to help clinicians in the direction provided by this research.

## Figures and Tables

**Table 1:** Scores for training and test set for the classification task.

	Training Set		Test Set					
	**N° Features**	**Accuracy** **(%)**	**Accuracy** **(%)**	**Recall** **(%)**	**Precision** **(%)**	**Sensitivity** **(%)**	**Specificity** **(%)**	**AUCROC**
RF	9	91.7	78.6	71.4	83.3	71.4	85.7	0.735
GB	22	75.0	92.9	85.7	100	85.0	100	0.857

RF = Random Forests, GB = Gradient Boosting, AUCROC = Area Under the Curve Receiver Operating Characteristics.

**Table 2:** Features chosen by Wrapper for the classification task.

RF	GB	
Base of support (cm)	Base of support (cm)	Stop Healthy VM
BMD of the Proximal region of femur	Toe In Out Operated (angle)	Start Healthy RFe
SD of the BMD of the Proximal region of femur	Velocity (m/s)	Stop Healthy RFe
VM Operated HU	Healthy leg BMD	Start Healthy VL
VM Healthy HU	RFe Operated HU	Stop Healthy VL
Stop Healthy VM	RFe Operated Dev. Std.	Start Operated VM
Stop Healthy RFe	RFe Healthy Dev. Std.	Stop Operated VM
Start Healthy VL	VL Operated HU	Start Operated RFe
Start Operated VL	VL Healthy HU	Stop Operated RFe
	VM Operated HU	Start Operated VL
	Start Healthy VM	Stop Operated VL

VM = Vastus Medialis, RFe = Rectus Femoris, VL = Vastus Lateralis, HU = Hounsfield Unit.

**Table 3:** Scores for the regression task.

		R^2^	Mean Absolute Error	Mean Squared Error	Root Mean Squared Deviation	Mean Signed Difference
Proximal BMD	RF	0.634	0.017	0.001	0.026	−0.004
	GB	0.539	0.018	0.001	0.029	−0.007
Distal BMD	RF	0.591	0.02	0.001	0.029	0.002
	GB	0.621	0.016	0.001	0.024	−0.004

RF = Random Forests, GB = Gradient Boosting.

**Table 4:** Features chosen through the correlation analysis.

Features	
Type of prosthesis	Start Healthy VM
Base of support (cm)	Stop Healthy VM
Double Support (% of gait cycle)	Start Healthy VL
Toe In Out Healthy (angle)	Stop Healthy VL
Toe In Out Operated (angle)	Stop Operated VM
SD of the BMD of the Proximal region of femur	Start Operated RFe
Healthy leg BMD	Stop Operated RFe
RFe Operated HU	Start Operated VL
VL Operated HU	Stop Operated VL
